# Endothelial Dysfunction in Nonalcoholic Fatty Liver Disease: A Systematic Review and Meta-Analysis

**DOI:** 10.3390/life12050718

**Published:** 2022-05-11

**Authors:** Panagiotis Theofilis, Aikaterini Vordoni, Nikolaos Nakas, Rigas G. Kalaitzidis

**Affiliations:** 1 Center for Nephrology “G. Papadakis”, General Hospital of Nikaia-Piraeus Agios Panteleimon, 18454 Piraeus, Greece; katerinavord@gmail.com (A.V.); rigaska@gmail.com (R.G.K.); 2 2nd Cardiology Department, General Hospital of Nikaia-Piraeus Agios Panteleimon, 18454 Piraeus, Greece; nksvnks@gmail.com

**Keywords:** nonalcoholic fatty liver disease, nonalcoholic steatohepatitis, flow-mediated dilation, endothelial dysfunction

## Abstract

Individuals with nonalcoholic fatty liver disease (NAFLD) are characterized by increased cardiovascular risk. Endothelial dysfunction, a mechanism implicated in those processes, may constitute the missing link in this interaction. Therefore, this systematic review and meta-analysis aims to evaluate the association of endothelial dysfunction, assessed by flow-mediated dilation (FMD) of the brachial artery, with NAFLD. We conducted a systematic literature search for studies assessing the difference in FMD between patients with NAFLD and controls. Exclusion criteria consisted of preclinical studies, studies in children/adolescents, no FMD assessment, and the absence of an NAFLD/control group. The database search identified 96 studies. Following the application of the exclusion criteria, 22 studies were included in the meta-analysis (NAFLD: 2164 subjects; control: 3322 subjects). Compared with controls, patients with NAFLD had significantly lower FMD% values (SMD: −1.37, 95% CI −1.91 to −0.83, *p* < 0.001, I^2^: 98%). Results remained unaffected after exclusion of any single study. Subgroup analysis revealed significantly decreased FMD in NAFLD subjects diagnosed with liver ultrasound or liver biopsy compared with method combination or other methods, while no differences were observed according to the chosen cuff inflation threshold, the presence of a significant difference in obesity measures between the groups, or the type of the control group (age- and sex-matched vs. other). Funnel plot asymmetry was not observed. Finally, compared with patients with pure steatosis, individuals with nonalcoholic steatohepatitis had significantly lower FMD (SMD: −0.81, 95% CI −1.51 to −0.31, *p* = 0.003, I^2^: 81%). In conclusion, FMD of the brachial artery, indicative of endothelial dysfunction, was significantly reduced in subjects with nonalcoholic fatty liver disease. Patients with nonalcoholic steatohepatitis might be facing a more pronounced endothelial impairment.

## 1. Introduction

Nonalcoholic fatty liver disease (NAFLD) is a heterogeneous entity with rising incidence worldwide, as evidenced by reported contemporary epidemiologic trends [1]. This may have important public health implications because it is associated with significant morbidity and mortality, which have risen in recent years [2]. Nonalcoholic steatohepatitis (NASH), a more advanced form of the disease confirmed by liver biopsy, may result in exceeding mortality rates [3]. Among the known risk factors for the development of NAFLD are the male sex, components of the metabolic syndrome (increased body mass index, hypertriglyceridemia, hyperglycemia, arterial hypertension), and hyperuricemia [4].

NAFLD terminology has been recently questioned, as experts presented significant drawbacks of this entity [5]. To begin with, NAFLD was considered a diagnosis of exclusion based on alcohol intake and the presence of viral hepatitis or autoimmune liver diseases. It should be made clear that the pathophysiology underlying NAFLD is frequently coexisting with the entities mentioned above, especially in individuals of developed countries. Moreover, determining alcohol consumption based on questionnaires is subjective, and setting safe alcohol intake limits is often debatable. Thus, the connection between fatty liver disease associated with metabolic disturbances and alcohol intake may be inappropriate. It should be also stressed that liver fibrosis, which has important prognostic implications, needs to be staged instead of being dichotomized to NASH or non-NASH. Finally, the management of the underlying pathophysiology is critical in the therapeutic approach of patients with NAFLD, as this is frequently heterogeneous. Ultimately, a new terminology by the name of metabolic associated fatty liver disease (MAFLD) has been instituted [5].

Importantly, NAFLD and NASH appear to confer a higher risk of incident cardiovascular events, independently of their known risk factors, as noted by the latest systematic review and meta-analysis conducted by Montovani et al. [6]. Moreover, advanced NAFLD could also predispose to the incidence of heart failure, leading to higher rates of hospitalizations, and all-cause and cardiovascular mortality [7]. Other than the shared risk factors, several pathophysiological mechanisms associating NAFLD to the incidence of cardiovascular diseases have been described. These include vascular inflammation, promotion of a prothrombotic state, dysregulated gut microbiota, and genetic or epigenetic modifications [8]. Additionally, the importance of endothelial dysfunction in this interplay appears to be critical. The development of NAFLD is accompanied by deleterious processes that may predispose to endothelial dysfunction, namely lipotoxicity, inflammation, oxidative stress, and apoptosis [9]. With that in mind, we have conducted a systematic review and meta-analysis to determine the presence of endothelial impairment, assessed by the flow-mediated dilation (FMD) of the brachial artery, in patients with NAFLD/NASH compared to controls.

## 2. Materials and Methods

### 2.1. Search Strategy and Inclusion and Exclusion Criteria

This systematic review and meta-analysis was conducted in accordance to the guidelines of the 2020 Preferred Reporting Items of Systematic Reviews and Meta-Analyses (PRISMA) statement [10], as shown in Table S1. The study was pre-registered in the PROSPERO International prospective register of systematic reviews (registration number: CRD42022318539).

We performed a literature search in PubMed from inception to 27 February 2022 in order to detect studies assessing the brachial artery FMD in patients with NAFLD and a control group. The following search terms were used: (“NAFLD” OR “fatty liver” OR “nonalcoholic fatty liver disease” OR “non-alcoholic fatty liver disease” OR “non-alcoholic steatohepatitis” OR “NASH”) AND (“flow-mediated dilation” OR “flow-mediated vasodilation” OR “FMD” OR “FMV” OR “endothelium-dependent dilation” OR “endothelial-dependent dilation” OR “endothelium-dependent vasodilation” OR “endothelial-dependent vasodilation”). The difference between the FMD of NAFLD patients and the control group was the primary outcome of interest. We excluded studies performed in preclinical models, children, or adolescents. Moreover, studies not using brachial FMD as the endothelial function assessment method, as well as those lacking an NAFLD group and/or a control group, were also excluded.

### 2.2. Data Extraction and Quality Assessment

The full text of the eligible studies was assessed by two independent review authors (P.T. and A.V.), who then proceeded to the data extraction that consisted of the FMD value (in %) in the NAFLD and the control group, publication year, method of NAFLD diagnosis (liver ultrasonography, liver biopsy, other, combination), cuff inflation threshold during the FMD measurement procedure, study group features (NAFLD or NASH, number of subjects, presence of a significant difference in obesity vs. controls, FMD value), and control group features (risk factor characteristics, number of subjects, FMD value). Consequently, the extracted data was cross-checked in a meeting. In the case of discrepancies in the data extraction, a third review author (R.G.K.), blinded to the initial data, was responsible for the reevaluation of the studies in question and making the final decision. Whenever significant information regarding FMD values was not included in the articles, we contacted corresponding authors via email. However, no replies were received after an attempt to contact the authors of two papers.

Numerical values of data represented graphically were extrapolated using Adobe Photoshop CS6 whenever they were not reported in the text. All numerical continuous data were transformed to mean ± standard deviation for the final analysis, as previously described [11]. Moreover, calculation of the overall FMD value in the presence of multiple NAFLD categories was performed [11]. The Joanna Briggs Institute (JBI) Critical Appraisal Checklist for the assessment of the methodological quality of studies was used as risk of bias tool [12].

### 2.3. Statistical Analysis

We performed a meta-analysis to assess the difference in brachial FMD between individuals with NAFLD and a control group. Effect sizes were pooled via random-effect model and the results are expressed as uncorrected standardized mean difference (SMD), using the Cohen’s d as the effect size metric, with 95% confidence intervals (CI). Between-study heterogeneity was assessed through the calculation of I^2^, with values of 25%, 50%, and 75% indicating mild, moderate, and substantial heterogeneity, respectively. A sensitivity analysis was conducted using the leave-one-out method and we additionally performed an influence analysis followed by an updated meta-analysis with the exclusion of the influential studies. Furthermore, graphic display of study heterogeneity (GOSH) plots were created, consisting of a combinatorial meta-analysis including 2^k−1^ analyses, with k representing the number of interventions. By using a κ-means algorithm and the Cook’s distance, studies were considered influential in cases where their Cook’s distance was over the calculated threshold. An updated meta-analysis was consequently conducted.

The existence of publication bias was assessed by funnel plot inspection and Egger’s test. Furthermore, we carried out a subgroup analysis according to NAFLD diagnostic method, cuff inflation threshold, the presence of age- and sex-matched controls, and the presence of a significant difference in obesity prevalence across the examined groups. Last but not least, a meta-analysis of studies presenting FMD values in an NAFLD (pure steatosis) group and a NASH group was also performed, to assess the difference in FMD according to NAFLD severity. *p* values of less than 0.05 signified statistical significance. All meta-analyses were generated using the meta and dmetar packages in R studio v.1.4.1106.

## 3. Results

### 3.1. Study Selection

**Figure 1:**
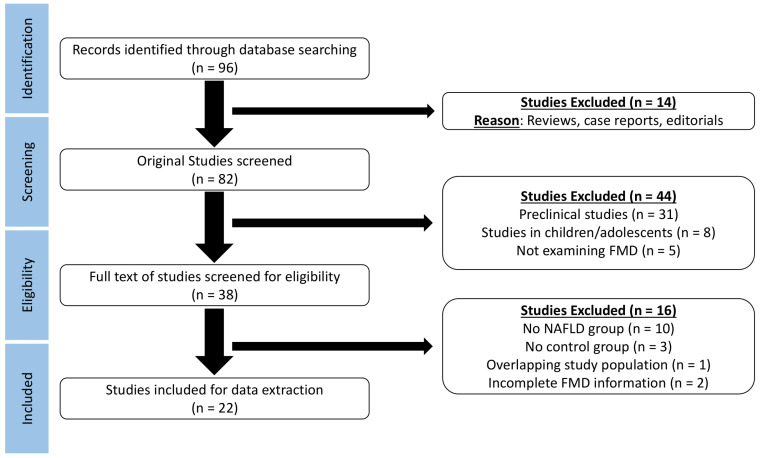
Preferred Reporting Items of Systematic Reviews and Meta-Analyses (PRISMA) flow diagram demonstrating the process of study selection in the meta-analysis.

Database search provided 96 results (Figure 1). After exclusion of reviews, case reports, and editorials, 82 studies were screened for eligibility. Exclusion of preclinical studies, studies in children or adolescents, and studies that did not assess brachial FMD lead to full text assessment of 38 studies. The lack of an NAFLD or control group, the overlapping study populations, and the incomplete FMD information (without a response from the corresponding author) led to further exclusion of 16 studies. Finally, 22 studies were considered for data extraction.

### 3.2. Study Characteristics

The characteristics of the included studies are presented in Table 1. From the 22 included studies, we identified 2164 individuals with NAFLD and 3322 control subjects. NAFLD diagnosis was made through liver ultrasound (8/22), liver biopsy (7/22), and method combination (5/22). One study used multi-detector abdominal computed tomography, while another utilized magnetic resonance spectroscopy. Regarding cuff inflation thresholds, 13 studies used a set value [200 mmHg (4/13), 220 mmHg (1/13), 250 mmHg (8/13)] and 5 studies inflated the cuff at 50 mmHg above the systolic blood pressure. Four studies did not report information on cuff inflation. As far as the NAFLD groups are concerned, the presence of NASH was assessed in 7 studies. In most of the studies (18/22) a significantly higher prevalence of obesity measures was reported in the NAFLD group compared with the control group. Age- and sex- matched control groups were present in 10 studies, 2 studies used subjects with chronic hepatitis B or C infection, while postmenopausal women were the control group in another study. Non-specific characteristics for the control group were present in six studies.

### 3.3. Meta-Analysis

**Figure 2:**
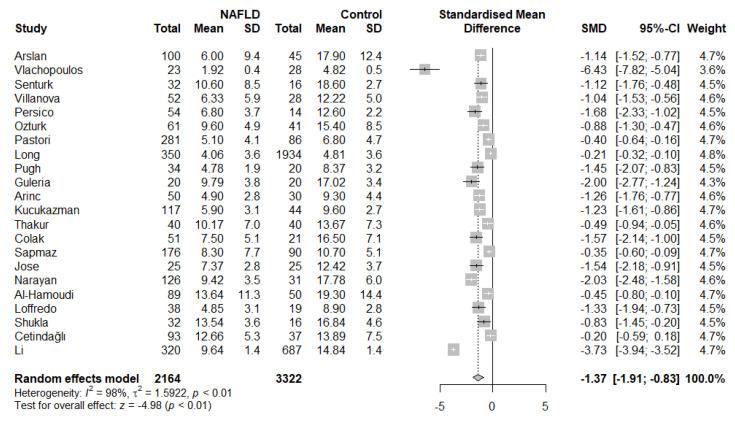
Forest plot displaying the meta-analysis of FMD difference between individuals with NAFLD and controls, demonstrating a significantly more impaired FMD in the NAFLD group. Effect sizes were pooled according to the random-effects model. I^2^ was used as a measure of between-study statistical heterogeneity. Results are expressed as standardized mean difference (SMD) with horizontal error bars denoting the 95% confidence intervals (CIs). The size of each square represents the relative weight of that study in the overall meta-analytic result.

**Figure 3:**
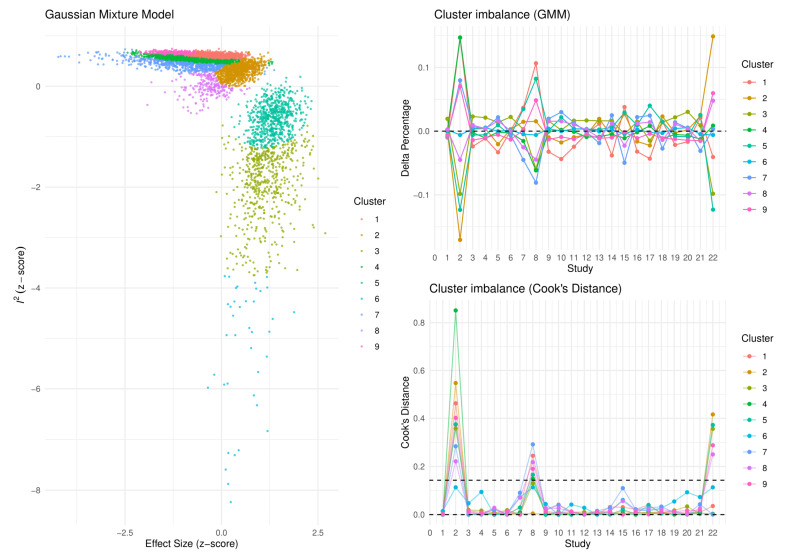
Graphic display of study heterogeneity (GOSH) plots of FMD difference between NAFLD and control subjects, showing the clusters that act as influential outliers towards between-study heterogeneity (I^2^) and overall effect size. A combinatorial meta-analysis was performed, including 2^k−1^ analyses, with k representing the number of interventions. The summary effects of those meta-analysis models (horizontal axis) and the heterogeneity (vertical axis) were illustrated graphically. Studies were considered influential in case their Cook’s distance was over the calculated threshold.

Based on the results of our meta-analysis, individuals with NAFLD had significantly more impaired brachial FMD compared with the respective controls (SMD: −1.37, 95% CI −1.91 to −0.83, *p* < 0.001) (Figure 2). Substantial between-study heterogeneity was noted (I^2^ = 98%). Omission of any single study did not alter the outcome of the results (Figure S1), while the exclusion of all the influential studies resulted in lower between-study heterogeneity (I^2^ = 61%) with little influence on the overall effect (SMD: −1.28, 95% CI −1.50 to −1.05, *p* < 0.001) (Figure S2). GOSH plots were also created to assess the presence of influential study clusters (Figure 3). After exclusion of influential studies [14,20,34], results remained largely unaffected (SMD: −1.06, 95% CI −1.32 to −0.81, *p* < 0.001, I^2^ = 84%). No indication of asymmetry was present upon funnel plot inspection and Egger’s regression test (intercept: −3.834, 95% CI −9.08 to 1.431, *p* = 0.17) (Figure S3). Most of the included studies were at an overall low risk of bias, with some studies not addressing the confounding factors (Table 2).

**Figure 4:**
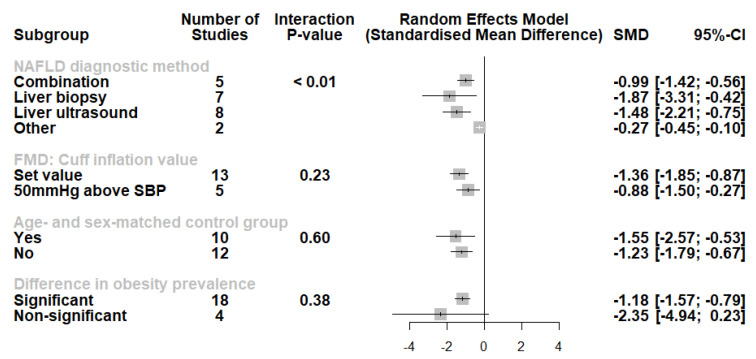
Subgroup analysis displaying no differences according to FMD cuff inflation threshold, the presence of age- and sex-matched control group, or a significant difference in obesity prevalence. However, we observed lowered effect sizes in studies using other NAFLD diagnostic methods (multi-detector abdominal computed tomography, magnetic resonance spectroscopy). Effect sizes were pooled according to the random-effects model and the subgroup analysis followed the fixed-effects (plural) model. Results are expressed as standardized mean difference (SMD) with horizontal error bars denoting the 95% confidence intervals (CIs).

**Figure 5:**
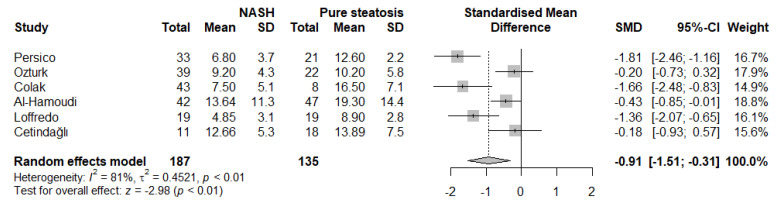
Forest plot displaying the meta-analysis of FMD difference between individuals with NASH and pure steatosis, demonstrating a significantly more impaired FMD in the NASH group. Effect sizes were pooled according to the random-effects model. I2 was used as a measure of between-study statistical heterogeneity. Results are expressed as standardized mean difference (SMD) with hori-zontal error bars denoting the 95% confidence intervals (CIs). The size of each square represents the relative weight of that study in the overall meta-analytic result.

A subgroup analysis was also conducted (Figure 4). Studies using liver biopsy and liver ultrasound had higher effect sizes compared with other methods or method combinations. Moreover, we noted no difference in the studies using set cuff inflation threshold or based on the systolic blood pressure of the participants (*p* for interaction = 0.23). When categorizing the studies according to the presence of an age- and sex-matched control group, no significant differences were observed (*p* for interaction = 0.60). Furthermore, although studies where nonsignificant differences in obesity measures had a lower effect size, this did not reach statistical significance (*p* for interaction = 0.38). Finally, we performed an analysis of studies involving participants with either histologically confirmed NASH or pure steatosis (Figure 5). Based on these results, individuals with NASH had significantly lower FMD values compared with those with pure steatosis (SMD: −0.91, 95% CI −1.51 to −0.31, *p* = 0.003, I^2^ = 81%).

## 4. Discussion

Through this systematic review and meta-analysis, based on 22 studies in adult humans, we tried to explore the association of NAFLD with endothelial dysfunction, a common pathophysiologic mechanism in cardiovascular diseases. Our results confirmed the hypothesis, highlighting the link between NAFLD and endothelial dysfunction, assessed via FMD of the brachial artery. Despite the substantial between-study heterogeneity that was noted, results were robust even after multiple sensitivity analyses. Moreover, the advanced form of the disease, NASH, may be associated with an even more severe endothelial impairment.

NAFLD represents a pathologic state with increasing incidence rates across the past decades. Initially evident as pure steatosis, NAFLD may progress to NASH and later to liver fibrosis. Diagnosis of NAFLD is based mostly on ultrasound investigation of the liver or characteristic histologic changes in the absence of notable alcohol consumption or other etiologies of chronic liver diseases [35]. Risk scores consisting of clinical and biochemical data have also been developed. Among them, the fatty liver index has a remarkable diagnostic accuracy according to the results of a large-scale, population-based study [36]. However, screening for NAFLD may be unnecessary since there are uncertainties regarding the long-term benefits of such practice, given the lack of appropriate therapeutic options.

Frequent reevaluation of patients with NAFLD is important, since a significant proportion will develop NASH and fibrosis, while others may exhibit disease regression [35]. The timeline of this progression is unpredictable since patients might present with advanced fibrosis even in the span of few years [35]. The most fearsome complication of NAFLD is the development of liver cirrhosis and hepatocellular carcinoma. Important genetic and ethnic prognostic considerations should also be taken into account. Concerning potential treatment options, lifestyle modifications remain the cornerstone of its management [35]. Additionally, pharmacological approaches may be considered with low quality of evidence. These may include antioxidant, anti-inflammatory, antiobesity, and antifibrotic medications, among others [35].

The components of metabolic syndrome are the most frequent risk factors associated with its occurrence. This fact has urged experts to reconsider the previously set terminology in order to stress the metabolic abnormalities that surround it. Therefore, future studies are needed to unveil novel diagnostic criteria and therapeutic interventions towards the MAFLD [5]. The underlying pathophysiology is believed to be mostly shared with its associated risk factors. Among the complex pathways involved, endothelial dysfunction appears to be a key process with deleterious outcomes.

Concerning the cardiovascular complications, a recently reported meta-analysis of 34,043 patients presented a 64% higher risk of developing major adverse cardiovascular events for NAFLD patients compared with those without NAFLD, with a median follow-up of 7 years [37]. The severity of NAFLD was associated with increasing cardiovascular morbidity and mortality. These findings were not confirmed in another meta-analysis, which could be attributed to the differences in methodological approaches [38]. However, common ground was identified, as NAFLD was associated with incident cardiovascular disease and coronary heart disease in particular [38]. At the same time, NASH was related to a significantly higher cardiovascular risk [38]. It should be noted that NAFLD is an independent predictor of vulnerable plaque presence, which may justify the increased risk for adverse cardiovascular events [39]. Moreover, in the case of incident acute coronary syndrome, the presence of NAFLD was predictive of higher in-hospital and long-term mortality rates [40].

The relationship between NAFLD and endothelial dysfunction may be important regarding the cardiovascular manifestations of NAFLD patients. This metabolic liver disease might be an independent aggravating factor towards endothelial impairment [41]. Such an effect could be driven by the increased low grade sterile inflammatory burden induced by NAFLD. The vascular endothelium, although initially believed to be a single layer lining the blood vessels, is now considered an abundant organ which regulates crucial homeostatic functions, including the regulation of vascular tone, the control of hemostasis, and vascular integrity [42]. Therefore, in the setting of endothelial cell dysfunction, those beneficial effects may be lost, leading to an adverse prognosis in patients with cardiovascular diseases [43]. Endothelial dysfunction is a frequent finding in patients with traditional cardiovascular risk factors, such as arterial hypertension, diabetes mellitus, dyslipidemia, obesity, and smoking [44]. It is often characterized as a precursor of atherosclerosis development and progression [45]. Several methods of endothelial function estimation have been proposed, with FMD of the brachial artery being the most widely used, mostly for research purposes. FMD has been associated with an increased incidence of adverse cardiovascular events and cardiovascular mortality [43]. Novel endothelial biomarkers have also been identified, namely endothelial microparticles, endocan, and soluble endoglin [46].

An inverse association between endothelial dysfunction and NAFLD has also been suggested. Preexisting endothelial impairment could promote metabolic hepatopathy owing to lower nitric oxide bioavailability and, consequently, hepatic stellate cell activation and sinusoidal thrombosis [41]. The role of liver sinusoidal endothelial cells in NAFLD may be critical in this direction, through their anti-inflammatory and antifibrotic properties [47]. Their dysfunction renders them unable to produce vasodilating substances in response to increased shear stress. Steatosis and development and progression continues under those conditions, and, in the stage of NASH, an additional release of proinflammatory mediators has been noted. Therefore, a vicious cycle involving stellate cell senescence and release of profibrotic molecules is being initiated, accompanied by angiogenesis. Liver fibrosis ensue with potential catastrophic hepatic complications such as liver cirrhosis and hepatocellular carcinoma. Thus, it becomes evident that maintenance of endothelial cell integrity across the different vascular beds could end up being essential in the prevention of NAFLD development and the attenuation of its progression. Ultimately, this may be translated in improved cardiovascular and hepatic morbidity and mortality.

The promotion of endothelial dysfunction in cases of NAFLD, as indicated by our study results, may mediate the excess cardiovascular-related morbidity and mortality. Thus, our study may have important implications, suggesting the evaluation of FMD in patients with NAFLD. Initially, ultrasonographic evaluation of steatosis, a cardinal feature of NAFLD, together with impaired FMD, could efficiently and reliably indicate a patient at an already increased risk for incident cardiovascular events who might benefit from early lifestyle modifications and pharmacological treatment for the strict control of risk factors. Moreover, adequate staging with imaging-based or histologic evaluation of hepatic inflammation and fibrosis represents an essential approach in high-risk patients with NAFLD [48]. Therefore, the presence of a significantly impaired FMD of the brachial artery could constitute a “red flag” for the presence of NASH or more advanced forms of the disease, mandating further investigation and potential inclusion in clinical trials of novel agents against NAFLD and NASH.

Several limitations of our study should be stressed. To begin with, the use of formulas for approximation of means and standard deviations from other summary measures, as well as the combination of means and standard deviations of different groups, may have had an influence on the overall findings. In addition, the overall results of our meta-analysis should be interpreted with caution on the basis of the considerable between-study heterogeneity. Sensitivity analyses attenuated the degree of heterogeneity to an extent. Moreover, the studies with nonsignificant differences in obesity measures were few, an observation which may have underestimated the importance of this factor in the NAFLD-related endothelial dysfunction.

## 5. Conclusions

Our systematic review and meta-analysis highlights the association of nonalcoholic fatty liver disease with endothelial dysfunction, assessed by the flow-mediated dilation of the brachial artery. Patients with nonalcoholic steatohepatitis might be facing a more pronounced endothelial impairment. Our findings provide the rationale for endothelial dysfunction screening in this patient population, since the degree of endothelial impairment might indicate an advanced form of the disease, related to an adverse hepatic and cardiovascular prognosis.

## Figures and Tables

**Table 1:** Characteristics of the included studies.

Study	Year	NAFLD Diagnosis	Cuff Inflation	Study Group			Control Group	
				Population	N	Difference in Obesity vs. Control	Population	N
Arslan [13]	2014	Biochemical, radiological, and histological criteria (when available)	250 mmHg	NAFLD	100	S	Healthy, age- and sex-matched	45
Vlachopoulos [14]	2010	Liver biopsy	NA	NAFLD	23	NS	Age-, gender-, BMI-, and CVRF-matched	28
Senturk [15]	2007	Liver biopsy	NA	NAFLD + NASH	32	S	Healthy	16
Villanova [16]	2005	Biochemical and radiological criteria (when available)	250 mmHg	NAFLD	52	S	Age- and sex-matched without metabolic diseases	28
Persico [17]	2017	Liver biopsy	250 mmHg	NAFLD + NASH	54	S	Healthy, age- and sex-matched	14
Ozturk [18]	2015	Liver biopsy	200 mmHg	NAFLD + NASH	61	S	Healthy	41
Pastori [19]	2015	Liver ultrasound	50 mmHg above SBP	NAFLD	281	S	NA	86
Long [20]	2015	Multi-detector abdominal CT	NA	NAFLD	350	S	NA	1934
Pugh [21]	2014	Magnetic resonance spectroscopy	220 mmHg	NAFLD	34	S	Obese	20
Guleria [22]	2013	Biochemical, radiological, and histological criteria (when available)	250 mmHg	NAFLD	20	S	Age- and sex-matched + chronic HBV/HCV	20
Arinc [23]	2013	Liver biopsy	50 mmHg above SBP	NASH	50	S	Healthy, age- and sex-matched	30
Kucukazman [24]	2013	Liver ultrasound	250 mmHg	NAFLD	117	S	NA	44
Thakur [25]	2012	Liver ultrasound	250 mmHg	NAFLD	40	NS	Healthy and age- and sex-matched	40
Colak [26]	2013	Liver biopsy	200 mmHg	NAFLD + NASH	51	S	Healthy	21
Sapmaz [27]	2016	Liver ultrasound	50 mmHg above SBP	NAFLD	176	S	NA	90
Jose [28]	2021	Liver ultrasound	250 mmHg	NAFLD	25	S	NA	25
Narayan [29]	2020	Liver ultrasound	250 mmHg	NAFLD	126	NS	HBV	31
Al-Hamoudi [30]	2020	Liver biopsy	200 mmHg	NAFLD + NASH	89	S	NA	50
Loffredo [31]	2018	Liver ultrasound ± biopsy	50 mmHg above SBP	NAFLD + NASH	38	S	Matched for age and relevant characteristics	19
Shukla [32]	2017	Liver ultrasound	NA	NAFLD	32	NS	Age- and sex-matched	16
Cetindağlı [33]	2017	Biochemical, radiological, and histological criteria (when available)	50 mmHg above SBP	NAFLD + NASH	93	S	Healthy and age- and sex-matched	37
Li [34]	2017	Liver ultrasound	200 mmHg	NAFLD	320	S	Postmenopausal women	687

NAFLD—nonalcoholic fatty liver disease; FMD—flow-mediated dilation; SBP—systolic blood pressure; CVRF—cardiovascular risk factors; NASH—nonalcoholic steatohepatitis; CT—computed tomography; HBV—hepatitis B virus; HCV—hepatitis C virus; NA—not available; S—significant; NS—nonsignificant.

**Table 2:** Assessment of risk of bias using the Joanna Briggs Institute (JBI) Critical Appraisal Checklist.

Study	Was the Criteria for Inclusion in the Sample Clearly Defined?	Were the Study Subjects and Setting Described in Detail?	Was the Exposure Measured in a Valid and Reliable Way?	Were Objective, Standard Criteria Used for Measurement of the Condition?	Were Confounding Factors Identified?	Were Strategies to Deal with Confounding Factors Stated?	Were the Outcomes Measured in a Valid and Reliable Way?	Were Appropriate Statistical Analysis Methods Used?
Arslan [13]	YES	YES	YES	YES	NO	NO	YES	YES
Vlachopoulos [14]	YES	YES	YES	YES	YES	YES	YES	YES
Senturk [15]	YES	YES	YES	YES	YES	NO	YES	YES
Villanova [16]	YES	YES	YES	YES	YES	YES	YES	YES
Persico [17]	YES	YES	YES	YES	YES	NO	YES	YES
Ozturk [18]	YES	YES	YES	YES	YES	YES	YES	YES
Pastori [19]	YES	YES	YES	YES	YES	YES	YES	YES
Long [20]	YES	YES	YES	YES	YES	YES	YES	YES
Pugh [21]	YES	YES	YES	YES	NO	NO	YES	YES
Guleria [22]	YES	NO	YES	YES	YES	NO	YES	YES
Arinc [23]	YES	YES	YES	YES	YES	YES	YES	YES
Kucukazman [24]	YES	YES	YES	YES	YES	YES	YES	YES
Thakur [25]	YES	YES	YES	YES	YES	YES	YES	YES
Colak [26]	YES	NO	YES	YES	YES	NO	YES	YES
Sapmaz [27]	YES	YES	YES	YES	YES	YES	YES	YES
Jose [28]	YES	YES	YES	YES	UNCLEAR	NO	UNCLEAR	UNCLEAR
Narayan [29]	YES	YES	YES	YES	YES	NO	YES	YES
Al-Hamoudi [30]	YES	YES	YES	YES	YES	NO	YES	YES
Loffredo [31]	YES	YES	YES	YES	YES	NO	YES	NO
Shukla [32]	YES	NO	UNCLEAR	UNCLEAR	NO	NO	UNCLEAR	NO
Cetindağlı [33]	YES	YES	YES	YES	YES	NO	YES	YES
Li [34]	YES	YES	YES	YES	YES	YES	YES	YES

## Data Availability

Not applicable.

## Supplementary Materials

The following supporting information can be downloaded at: https://www.mdpi.com/article/10.3390/life12050718/s1, Figure S1: Leave-one-out sensitivity analysis, ordered by effect size (θ), demonstrating that even after exclusion of any single study, the outcome of the meta-analysis concerning the difference in FMD between NAFLD and control subjects remained unaffected. I^2^ represents between-study heterogeneity and horizontal error bars denote the 95% confidence intervals, Figure S2: Forest plot displaying the updated meta-analysis of FMD difference between individuals with NAFLD and controls after removal of influential studies, demonstrating a significantly more impaired FMD in the NAFLD group. Effect sizes were pooled according to the random-effects model. I^2^ was used as a measure of between-study statistical heterogeneity. Results are expressed as standardized mean difference (SMD) with horizontal error bars denoting the 95% confidence intervals (CIs). The size of each square represents the relative weight of that study in the overall meta-analytic result, Figure S3: Inspection of symmetric funnel plots of the difference in FMD between NAFLD and control subjects. Hedge’s g was used as the effect size metric plotted against the sample size-based precision estimate, Table S1: Preferred Reporting Items of Systematic Reviews and Meta-Analyses (PRISMA) statement checklist.
