# Supplementary material for: Endothelial Dysfunction in Nonalcoholic Fatty Liver Disease: A Systematic Review and Meta-Analysis
Source: Life (Basel). 2022 May 11;12(5):718. doi: 10.3390/life12050718 (PMC9144621; doi:10.3390/life12050718)
Supplement: Supplementary file 1 [file life-12-00718-s001.zip › Supplementary Figures S1-S3.pdf]

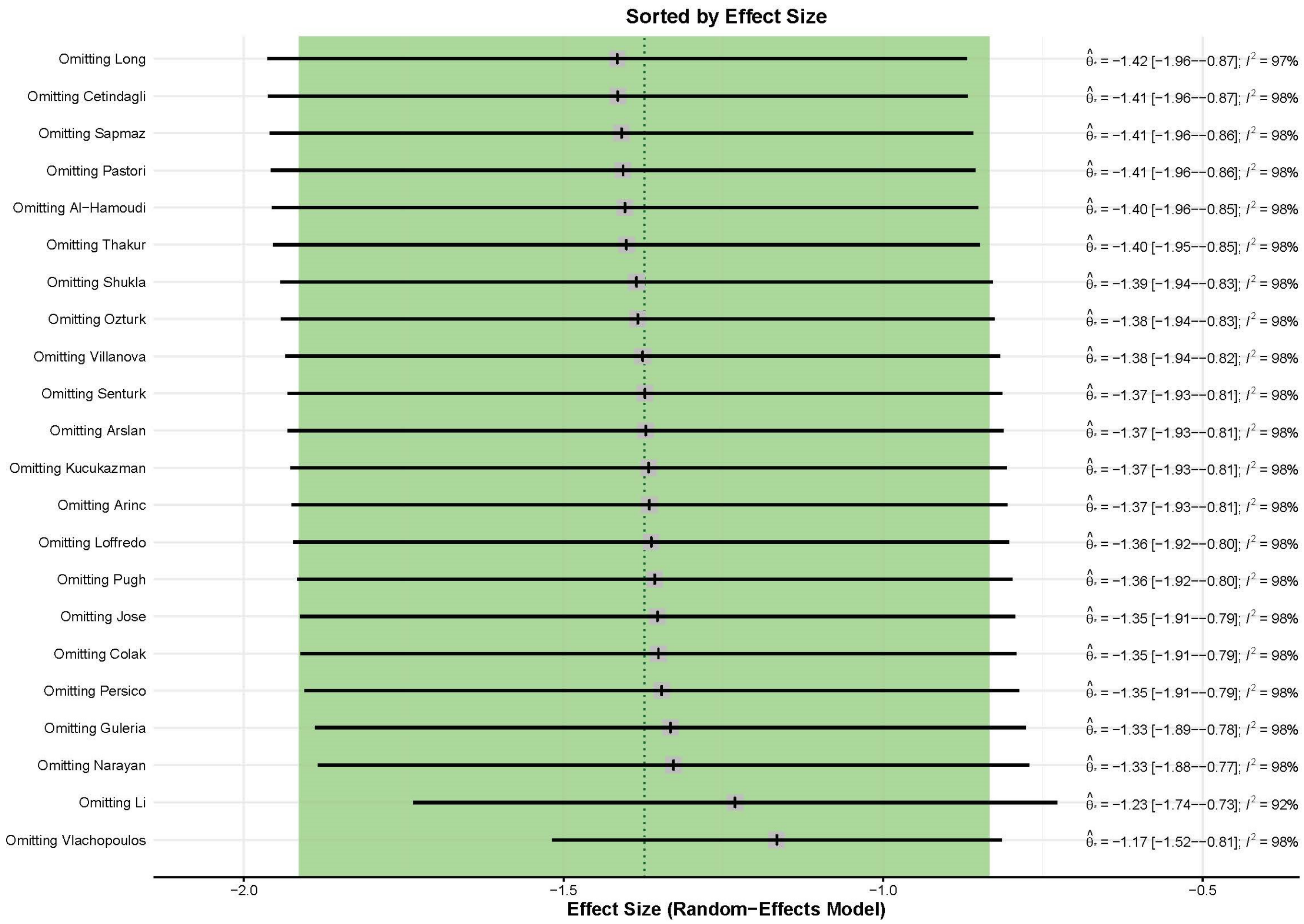

Figure S1. Leave-one-out sensitivity analysis, ordered by effect size (θ), demonstrating that even after exclusion of any single study, the outcome of the meta-analysis concerning the difference in FMD between NAFLD and control subjects remained unaffected. I^2 represents between-study heterogeneity and horizontal error bars denote the 95% confidence intervals.

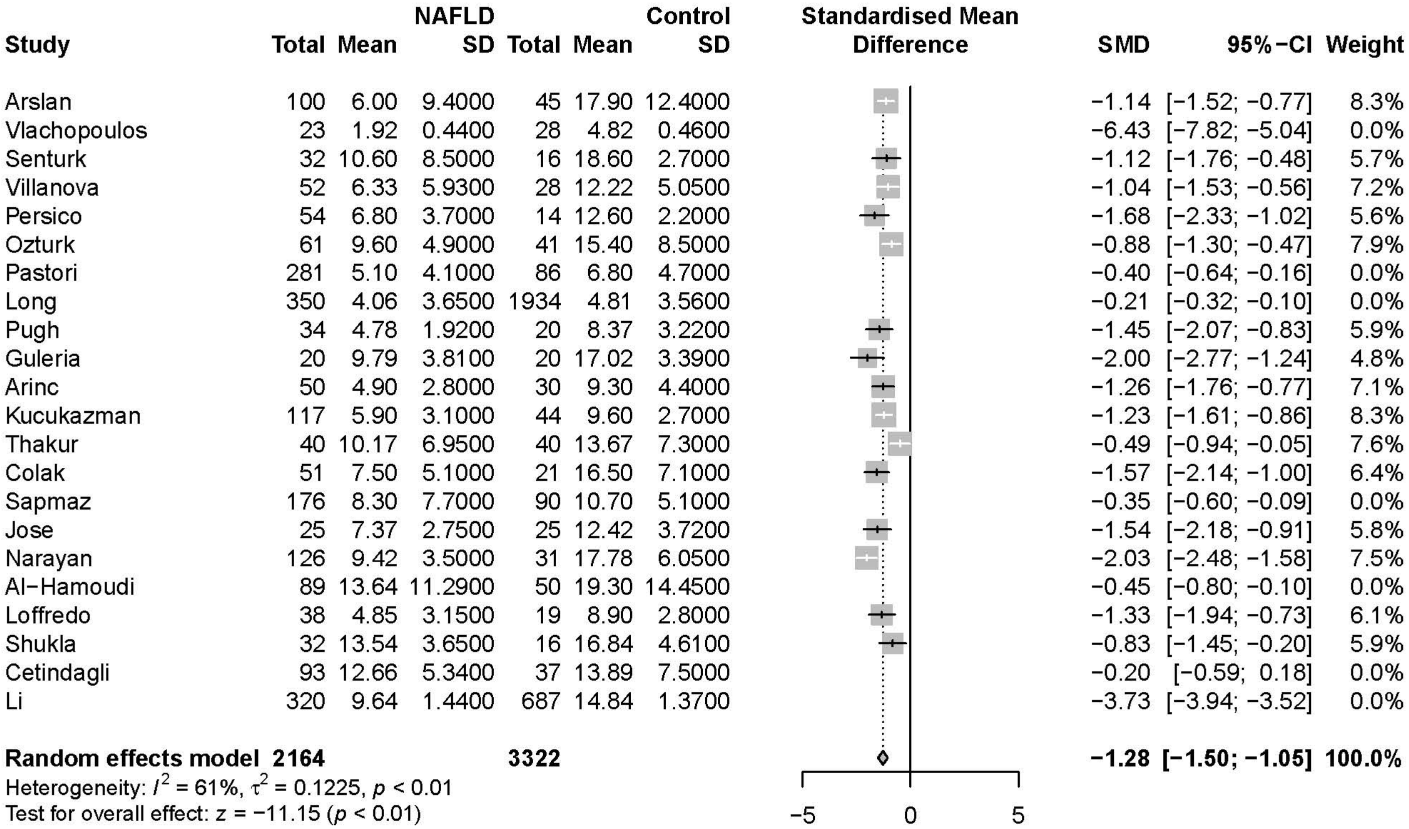

Figure S2. Forest plot displaying the updated meta-analysis of FMD difference between individuals with NAFLD and controls after removal of influential studies, demonstrating a significantly more impaired FMD in the NAFLD group. Effect sizes were pooled according to the random-effects model. I^2 was used as a measure of between-study statistical heterogeneity. Results are expressed as standardized mean difference (SMD) with horizontal error bars denoting the 95% confidence intervals (CIs). The size of each square represents the relative weight of that study in the overall meta-analytic result.

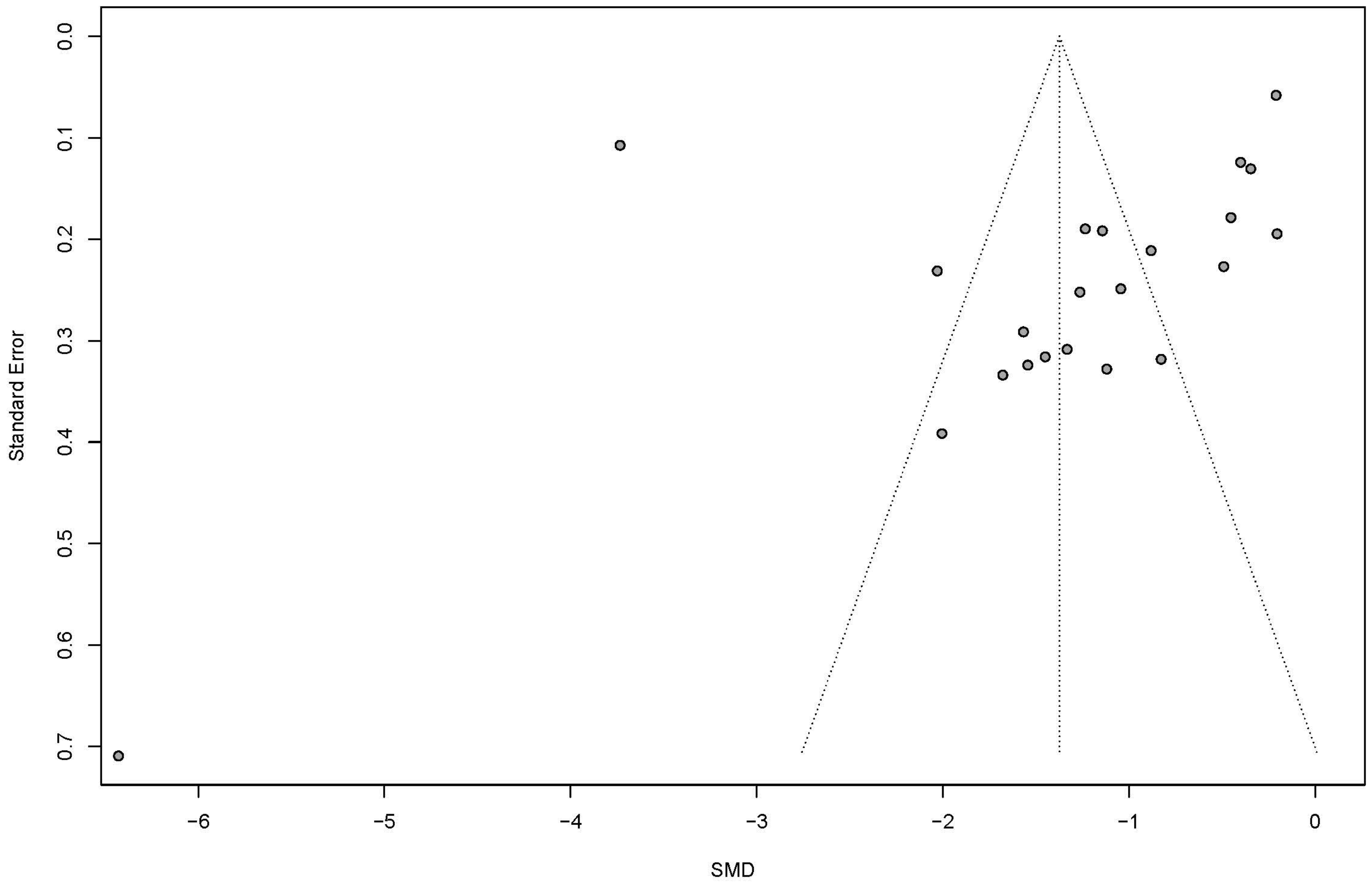

Figure S3. Inspection of symmetric funnel plots of the difference in FMD between NAFLD and control subjects. Hedge's g was used as the effect size metric plotted against the sample size-based precision estimate.
